# Rural-Urban Differences in Prevalence and Associated Factors of Underweight and Overweight/Obesity among Bangladeshi Adults: Evidence from Bangladesh Demographic and Health Survey 2017–2018

**DOI:** 10.3390/epidemiologia4040042

**Published:** 2023-11-22

**Authors:** Rajat Das Gupta, Hanna A. Frank, Maxwell Akonde, Ananna Mazumder, Nazeeba Siddika, Ehsanul Hoque Apu, Promit Ananyo Chakraborty

**Affiliations:** 1 Department of Epidemiology and Biostatistics, Arnold School of Public Health, University of South Carolina, Columbia, SC 29208, USA; 2 School of Population and Public Health, University of British Columbia, Vancouver, BC V5Z 1M9, Canada; 3 Jahurul Islam Medical College (JIMC), Bajitpur, Kishoreganj 2336, Bangladesh; 4 Department of Epidemiology and Biostatistics, College of Human Medicine, Michigan State University, East Lansing, MI 48824, USA; 5 Centre for International Public Health and Environmental Research, Bangladesh (CIPHER,B), Dhaka 1207, Bangladesh; 6 Department of Biomedical Engineering, Institute of Quantitative Health Science and Engineering, Michigan State University, East Lansing, MI 48824, USA; 7 Department of Internal Medicine, Division of Hematology and Oncology, University of Michigan, Ann Arbor, MI 48105, USA; 8 Department of Biomedical Science, College of Dental Medicine, Lincoln Memorial University, Knoxville, TN 37923, USA

**Keywords:** underweight, overweight, obesity, Bangladesh

## Abstract

The aim of this study was to identify the differences in prevalence and associated factors of underweight and overweight/obesity among Bangladeshi adults (≥18 years) by analyzing the cross-sectional Bangladesh Demographic and Health Survey 2017–2018 data. Multilevel multivariable logistic regression was applied to identify the factors associated with underweight and overweight/obesity in urban and rural areas. The prevalence of underweight was 12.24% and 19.34% in urban and rural areas, respectively. The prevalence of overweight/obesity was 50.23% and 35.96%, respectively, in urban and rural areas. In the final multivariable analysis in both urban and rural areas, 30–49 years of age, female sex, being educated up to college or higher level, living in the wealthiest household, and being currently married or being separated/divorced/widowed had higher odds of being overweight/obese compared to other categories. Residence in the Mymensingh and Sylhet region was associated with decreased odds of overweight/obesity in urban and rural areas. On the other hand, being educated up to college or higher level, living in the wealthiest household, and being married were associated with reduced odds of being underweight in both areas. These high-risk groups should be brought under targeted health promotion programs to curb malnutrition.

## 1. Introduction

In the past few decades, the prevalence of overweight and obesity has surged, becoming a global health crisis [1,2]. Obesity is associated with non-communicable diseases, including type 2 diabetes mellitus, cardiovascular diseases, kidney diseases, chronic liver disease, osteoarthritis, and obstructive sleep apnea [1,3]. Obesity may also be associated with body dissatisfaction and impaired physical functioning, leading to depression and social isolation [3]. The higher prevalence of overweight and obesity has been observed in both high-income countries (HICs) and low- and middle-income countries (LMICs), though in HICs, the trends have recently flattened [4]. LMICs not only continue to face a rapidly increasing prevalence of obesity but also the added challenge of a high underweight prevalence [4,5,6]. Undernutrition, in particular, is associated with insulin resistance and lower fat oxidation, leading to a higher risk of diabetes, hypertension, dyslipidemia, and physiological impairments [7]. Therefore, LMICs must address two vastly different but comparably dangerous nutritional trends in their populations.

An examination of the 2014 Bangladesh Demographic and Health Survey (BDHS) data by Hashan et al. [5], revealed that around 21% of the rural women were underweighted. In contrast, more than half of urban women (53%) were overweight/obese. The study confirmed the presence of a ‘double burden’ of simultaneous high underweight and overweight prevalence in women of reproductive age in Bangladesh. It examined the factors associated with the risk of being underweight and overweight in this population [5]. However, much has changed in Bangladesh since the collection of the BDHS 2014 data. For instance, in 2014, Bangladesh’s gross domestic product (GDP) growth was 6.04%, which increased to 7.86% in 2018 [8]. This change in GDP, coupled with rapid urbanization, has hugely impacted the previously existing demography. Rapid urbanization is associated with economic development and contributes to inequalities and health-related issues [9].

Additionally, there has been an estimated increase in the number of refugees between 2014 and 2018, with an estimated one million refugees living in Bangladesh by mid-2018 [10]. Refugee populations often struggle with food insecurity and develop unhealthy nutritional habits along with a more sedentary lifestyle, and it has an impact on the host community, too [10], including increased prevalence of underweight- or overweight-related health conditions.

These changes in recent years could affect the prevalence of underweight and overweight in Bangladesh, potentially affecting rural and urban areas differently. Moreover, BDHS 2014 only collected anthropometric data of women of reproductive age group. It is, therefore, essential to analyze a more recent survey to examine trends and changes since the BDHS 2014 to inform further policy decisions. This study will examine the prevalence of underweight and overweight and the associated factors in the general population of Bangladesh, stratified by rural and urban living environments, using the latest BDHS 2017–2018 data. Exploring factors associated with the underweight and overweight prevalence is essential to target especially vulnerable groups and tailor measures taken to reduce the impacts of malnutrition. Socioeconomic factors are critical to reassess after significant changes in Bangladesh since the BDHS 2014. An updated analysis of the critical relationships using recent data can illuminate any changes in these relationships and further inform decisions about how to address the problem adequately. So far, there has not been a study examining these factors in the general population of Bangladesh with the new data. The study aims to identify the differences in prevalence and associated factors of underweight and overweight/obesity among Bangladeshi adults (≥18 years) using BDHS 2017–2018 data.

## 2. Materials and Methods

### 2.1. Study Design

This study utilized the BDHS 2017–2018 data, a nationwide cross-sectional survey to update the health-related indicators of children and adults in Bangladesh. Mitra and associates collected the data from October 2017 to October 2018. The authors obtained permission from the Demographic Health Survey (DHS) program to utilize the dataset for this study in December 2020. Data analysis was conducted between January 2021 and April 2021 [11].

**Figure 1:**
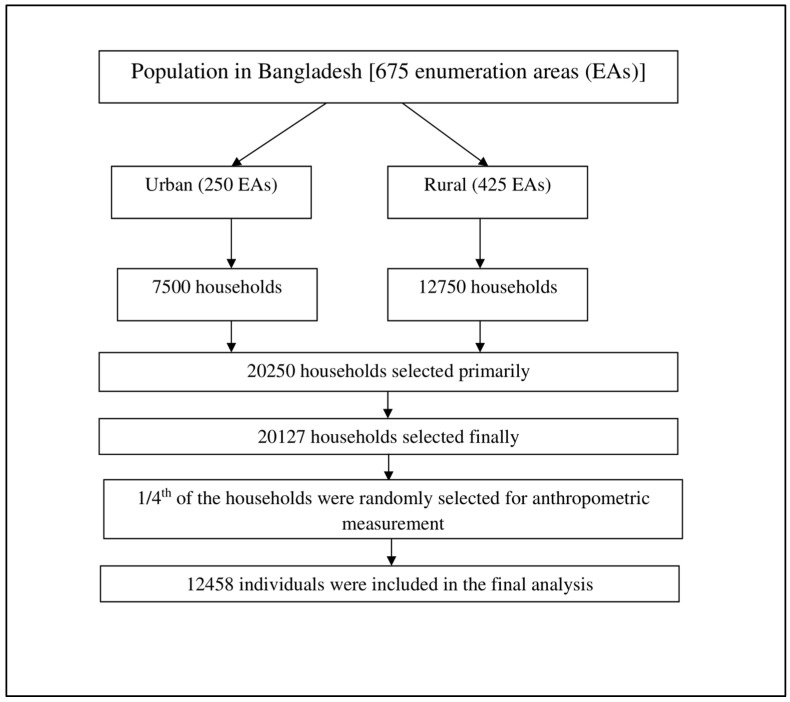
Flow diagram showing the selection process of participants from the 2017–2018 Bangladesh Demographic and Health Survey data.

A previous report described the survey design, methodologies, data collection tools, techniques, and process [11]. BDHS 2017–2018 utilized a two-stage stratified cluster sampling. First, enumeration areas (EAs) were selected (comprised of 120 households on average), which served as a sampling frame and primary sampling unit. In selecting EAs, the 2011 population and housing census of Bangladesh was used as the base of the sampling frame, and EAs were selected based on probability proportional to size method. In the urban area, 250 EAs were selected. In the rural area, 425 EAs were selected. Second, from each EA, 30 households were randomly selected. In total, 20,250 households were selected. From one-fourth of the selected households, all adults (aged ≥ 18 years) were selected for anthropometric measurement [11], who formed the sample for analysis in this study. The sample selection is shown in Figure 1.

### 2.2. Data Collection Tools

The 2017–2018 Bangladesh Demographic and Health Survey (BDHS) utilized six questionnaires for data collection: Household, Woman’s, Biomarker, Verbal Autopsy (two versions), Community, and Fieldworker. These tools were adapted from previous DHS and BDHS iterations, incorporating specific needs and contexts of Bangladesh. The Household Questionnaire identified eligible women for interviews and biomarker assessments, gathering information on individual and household characteristics. The Woman’s Questionnaire, aimed at ever-married women aged 15–49, covered a range of topics from reproductive history to husbands’ backgrounds. Biomarker data included measurements including height, weight, blood pressure, and blood glucose. The Verbal Autopsy questionnaires collected data on causes of death in children aged under 5 years. The Community Questionnaire provided insights into health services and facilities’ availability, while the Fieldworker Questionnaire helped in analyzing data quality. These tools were developed collaboratively, translated into Bangla, and underwent back translations to ensure accuracy [11]. ShorrBoard^®^ measuring board (Shorr production, Olney, MD, USA) in standing position and lightweight, electronic SECA 878 scale (seca Deutschland, Hamburg, Germany) were used to measure height and weight, respectively [11].

### 2.3. Data Collection

The questionnaires were pretested between 16 and 30 August 2017, by a team comprising four supervisory staff, 16 interviewers, and three personnel specializing in biomarkers. The pretesting was conducted in 100 households in both rural and urban settings. Subsequent to field observations and recommendations from the pretesting, modifications were implemented in the questionnaire design [11].

The training for household listers and mappers was conducted from 17 to 21 September 2017. There were two separate training sessions for the main survey. The first session focused on the Household and Woman’s Questionnaires and was attended by interviewers, team leaders, and quality control personnel. The second session covered biomarker aspects and was for health technicians. From 24 September to 22 October 2017, fieldworkers received their training, divided into four groups of approximately 50–55 individuals each. A total of 210 field staff, chosen based on education, experience, maturity, and a 4-month commitment to the project, participated. The training comprised lectures on questionnaire completion, practice interviews, and field exercises. Key survey staff and senior professionals from Mitra and Associates led the training sessions. Experts from ICF and National Institute of Population Research and Training (NIPORT) served as resource people, and a representative from the Ministry of Health and Family Welfare’s (MOHFW) Directorate General of Health Services delivered a presentation on the Expanded Program on Immunization and vaccines for infants and children. Additionally, staffs from the International Centre for Diarrhoeal Disease Research, Bangladesh (ICDDR,B) provided training on verbal autopsy questionnaires [11].

Field activities for the main survey were distributed over five stages, extending from 24 October 2017 to 15 March 2018. This process began with 20 teams and concluded with 17. Various quality assurance protocols were employed, including oversight visits from Mitra and Associates and NIPORT teams, random verifications, and the generation of weekly reports to detect and address any issues arising during the data collection phase [11].

The data processing phase for the BDHS questionnaires initiated in November 2017 and entailed a series of tasks such as in-office editing, coding, data entry, and meticulous verification for inconsistencies, culminating on 27 March 2018. The Census and Survey Processing System (CSPro) software was utilized for this data processing phase. Identified discrepancies or mistakes were systematically relayed to the field units for appropriate rectifications [11].

### 2.4. Outcome of Interest

The outcome of interest was body mass index (BMI). BMI was calculated by dividing an individual’s body weight in kilograms by the square of the height meters [11]. Asia-specific BMI cut-off was used to categorize the participants as underweight (<18.5 kg/m^2^), normal BMI (18.5 to <23.0 kg/m^2^), and overweight/obese (≥23.0 kg/m^2^) [12]. The comparison of World Health Organization (WHO)-recommended Body Mass Index (BMI) cut-off values with Asia-specific BMI cut-off values is shown in Table A1.

### 2.5. Explanatory Variables

Based on the literature review, as well as biological and social plausibility, the following explanatory variables were used: age (18–29 years, 30–49 years, 50–69 years, ≥70 years), sex (male, female), educational status (no formal education, primary, secondary, college and higher), wealth index (poorest, poorer, middle, richer, and richest), current working status (no, yes), division of residence (Barisal, Chattogram, Dhaka, Khulna, Mymensingh, Rajshahi, Rangpur, and Sylhet), and marital status (Never Married, Currently Married, Separated/Divorced/Widowed) [5,13,14]. The information about these variables was collected through questionnaires. BDHS 2017–2018 collected information on selected assets possession of the respondents (construction materials to build households, water source and sanitation facilities’ type, use of electricity, health services and other amenities). Principal component analysis was done to calculate the wealth index, which was then categorized into quintiles [11,15,16,17].

### 2.6. Statistical Analysis

In total, a sample of 12,458 participants was included. We conducted complete case analysis because of low frequency of missing values (<5%) [18]. A descriptive analysis was conducted, and the findings were presented in weighted frequencies and percentages according to urban–rural strata. The prevalence of both underweight and overweight was calculated. Chi-square test was conducted to determine the differences in the prevalence of BMI categories across independent variables. Multilevel logistics regression was conducted to determine the associated factors of underweight and overweight in urban and rural strata. During the analyses, normal BMI was considered as the reference category. The multilevel regression was conducted considering the hierarchical structure of the BDHS data. At first, crude logistic regression was conducted. Then, the variables that yielded a *p*-value < 0.2 were put in the multivariable model (which was considered enough to control residual confounding) [19]. Both crude odds ratios (COR) and adjusted odds ratios (AOR) were calculated and presented with 95% confidence interval (CI). A *p*-value < 0.05 was considered statistically significant.

Multivariable model for urban area outcomes were:

Log(Odds of Underweight) = β1*Age_30–49 + β2*Age_50–69 + β3*Age_70+ + β4*Education_Primary + β5*Education_Secondary + β6*Education_CollegeAndHigher + β7*WealthIndex_Poorer + β8*WealthIndex_Middle + β9*WealthIndex_Richer + β10*WealthIndex_Richest + β11*CurrentWorkingStatus_Yes + β12*DivisionOfResidence_Chattogram + β13*DivisionOfResidence_Dhaka + β14*DivisionOfResidence_Khulna + β15*DivisionOfResidence_Mymensingh + β16*DivisionOfResidence_Rajshahi + β17*DivisionOfResidence_Rangpur + β18*DivisionOfResidence_Sylhet + β19*MaritalStatus_CurrentlyMarried + β20*MaritalStatus_SeparatedDivorcedWidowed
Log(Odds of Overweight/Obesity) = β1*Age_30–49 + β2*Age_50–69 + β3*Age_70+ + β4*Sex_Female + β5*Education_Primary + β6*Education_Secondary + β7*Education_CollegeAndHigher + β8*WealthIndex_Poorer + β9*WealthIndex_Middle + β10*WealthIndex_Richer + β11*WealthIndex_Richest + β12*CurrentWorkingStatus_Yes + β13*DivisionOfResidence_Chattogram + β14*DivisionOfResidence_Dhaka + β15*DivisionOfResidence_Khulna + β16*DivisionOfResidence_Mymensingh + β17*DivisionOfResidence_Rajshahi + β18*DivisionOfResidence_Rangpur + β19*DivisionOfResidence_Sylhet + β20*MaritalStatus_CurrentlyMarried + β21*MaritalStatus_SeparatedDivorcedWidowed

Multivariable model for rural area outcomes were:

Log(Odds of Underweight) = γ1*Age_30–49 + γ2*Age_50–69 + γ3*Age_70+ + γ4*Education_Primary + γ5*Education_Secondary + γ6*Education_CollegeAndHigher + γ7*WealthIndex_Poorer + γ8*WealthIndex_Middle + γ9*WealthIndex_Richer + γ10*WealthIndex_Richest + γ11*DivisionOfResidence_Chattogram + γ12*DivisionOfResidence_Dhaka + γ13*DivisionOfResidence_Khulna + γ14*DivisionOfResidence_Mymensingh + γ15*DivisionOfResidence_Rajshahi + γ16*DivisionOfResidence_Rangpur + γ17*DivisionOfResidence_Sylhet + γ18*MaritalStatus_CurrentlyMarried + γ19*MaritalStatus_SeparatedDivorcedWidowed
Log(Odds of Overweight/Obesity) = γ1*Age_30–49 + γ2*Age_50–69 + γ3*Age_70+ + γ4*Sex_Female + γ5*Education_Primary + γ6*Education_Secondary + γ7*Education_CollegeAndHigher + γ8*WealthIndex_Poorer + γ9*WealthIndex_Middle + γ10*WealthIndex_Richer + γ11*WealthIndex_Richest + γ12*CurrentWorkingStatus_Yes + γ13*DivisionOfResidence_Chattogram + γ14*DivisionOfResidence_Dhaka + γ15*DivisionOfResidence_Khulna + γ16*DivisionOfResidence_Mymensingh + γ17*DivisionOfResidence_Rajshahi + γ18*DivisionOfResidence_Rangpur + γ19*DivisionOfResidence_Sylhet + γ20*MaritalStatus_CurrentlyMarried + γ21*MaritalStatus_SeparatedDivorcedWidowed

Data were analyzed with Stata V.16.0.

### 2.7. Ethical Consideration

The BDHS 2017–2018 study protocol was approved by the institutional review board at ICF (IRB: FWA00000845) and the Bangladesh Medical Research Council (IRB: BMRC/NREC/2016–2019/324). Informed consent was taken from the study participants prior to data collection. As the current study utilized a publicly accessible, de-identified dataset, it was exempted from ethical review and approval.

## 3. Results

### 3.1. Characteristics of the Study Sample

In general, the prevalence of underweight was 12.24% and 19.34% in urban and rural areas, respectively. On the other hand, the prevalence of overweight/obesity was 50.23% and 35.96%, respectively, in urban and rural areas.

The sample characteristics according to the prevalence of different categories of BMI in urban and rural regions are shown in Table 1. In both urban and rural areas, the prevalence of underweight was highest among the 18–29 years old age group (among all the age groups, urban: 18.7%, rural: 21.7%), male sex (between the sex category, urban: 14.2%, rural: 22.0%), received no formal education (among the education categories, urban: 16.2%, rural: 27.1%), poorest wealth quintile (among the wealth quintiles, urban: 24.2%, rural: 28.5%), currently working (between the current working status categories, urban: 12.7%, rural: 20.0%), and never married (among the marital status category, urban: 23.1%, rural: 26.4%). Among the division of residence, the prevalence of underweight was the highest in the Sylhet division in the urban area (19.8%) and in the Mymensingh division in the rural area (27.4%).

On the other hand, the prevalence of overweight/obesity was highest among the female sex (between the sex category, urban: 57.1%, rural: 41.4%), being educated up to college and higher (between the education categories, urban: 63.2%, rural: 45.0%), richest wealth quintile (among the wealth quintiles, urban: 63.4%, rural: 62.9%), not currently working (between the current working status categories, urban: 57.6%, rural: 39.2%), and currently married (among the marital status category, urban: 53.8%, rural: 38.5%). The prevalence was the highest among the 30–49 years old in the urban area (53.8%), and the ≥70 years old in the rural area (38.1%). Among the division of residence, the prevalence of overweight/obesity was the highest in the Khulna division in the urban area (53.7%) and in the Chattogram division in the rural area (44.6%).

The differences in the prevalence of underweight and overweight/obesity between urban and rural area according to categories are shown in Table A2 and Table A3, respectively. The prevalence of underweight was higher in rural areas compared to urban areas across all age groups, genders, education levels, and divisions of residence, with significant differences particularly noted in age groups 30–49, 50–69, and 70+ years, and among those with no formal schooling (Table A2). On the other hand, the prevalence of overweight/obesity was higher in urban areas, with significant differences observed across most categories including age groups, gender, education level, and working status, particularly among individuals aged 30–49 and 50–69 years, females, and those with higher education (Table A3). Socioeconomic factors such as wealth index and marital status showed varied impacts; while the prevalence of underweight does not significantly differ by wealth index (Table A1), the prevalence of overweight/obesity is higher among the richest compared to the poorest in urban areas (Table A2).

### 3.2. Factors Associated with Underweight

Factors associated with being underweight in both urban and rural areas among the adult Bangladeshi population are shown in Table 2. Education, wealth index, and marital status were significantly associated with underweight in both places. The odds of being underweight decreased with increasing education and wealth status. However, in the urban area, a statistically significant association was found only for those who received college and higher-level education. Being educated up to college and at a higher level decreased the odds of being underweight by 40% compared to those who did not receive any education (AOR:0.6; 95% CI: 0.4–0.9; *p* = 0.019). A similar decrease in the odds was also observed in the rural area (AOR:0.6; 95% CI: 0.5–0.8; *p* < 0.001). In both urban and rural areas, the odds of underweight were 50% less among the richest wealth quintile (AOR: Urban: 0.5; 95% CI: 0.4–0.8; *p* < 0.001; rural: 0.5; 95% CI: 0.3–0.6; *p* < 0.001) in comparison to the poorest wealth quintile. Compared to the never married women, currently married women were 40% less likely to be underweight in the urban area (AOR: 0.6; 95% CI: 0.4–0.7; *p* < 0.001), and 30% less likely to be underweight in the rural area (AOR: 0.7; 95% CI: 0.5–0.8; *p* < 0.001). In addition to that, age and current working status were associated with being underweight in the urban area. In the urban area, those aged 30–49 years were 40% less likely to be underweight than those aged 18–29 (AOR: 0.6; 95% CI: 0.4–0.8; *p* = 0.001). On the other hand, current workers had 20% lesser odds of being underweight than those who were not working (AOR: 0.8; 95% CI: 0.7–1.0, *p* = 0.045). On the other hand, division of residence was associated with underweight in the rural area. The residents of the rural Mymensingh division had 40% greater odds of being underweight than those of the rural Barisal division (AOR: 1.4; 95% CI: 1.1–1.8; *p* = 0.018).

### 3.3. Factors Associated with Overweight and Obesity

Factors associated with overweight/obesity in urban and rural areas among the adult Bangladeshi population are shown in Table 3. In both areas, age, sex, education, wealth index, division of residence, and marital status were associated with overweight/obesity. Increasing age was associated with an increased risk of overweight/obesity. In the rural area, being aged ≥ 70 years significantly increased the odds of overweight/obesity by 40% (AOR: 1.4; 95% CI: 1.1–1.8; *p* = 0.01) compared to those aged 18–29 years. No significant increase was observed for the urban counterparts (although a significant increase was observed for 30–49 years and 50–69 years). Compared to those aged 18–29 years, those aged 30–49 years had 40% higher odds (AOR: 1.4; 95% CI: 1.1–1.9; *p* = 0.004) in the urban area and 50% higher odds (AOR: 1.5; 95% CI: 1.2–1.8; *p* < 0.001) in the rural area. The odds were higher for females than males (AOR: Urban: 1.7; 95% CI: 1.4–2.0; *p* < 0.001; rural: 2.0; 95% CI: 1.8–2.3; *p* < 0.001). The odds of overweight/obesity increased with increasing education. Those educated up to college and higher level were twice as likely to be overweight/obese than those who received no formal education in the urban area (AOR: 2.1; 95% CI: 1.7–2.7; *p* < 0.001). For the rural counterparts, the increase in the odds of overweight/obesity was 70% (AOR: 1.7; 95% CI: 1.4–2.1; *p* < 0.001). Similarly, the odds of overweight/obesity increased with increasing wealth index. Those who belonged to the richest households were four times more likely to be overweight/obese compared to the poorest households (AOR: Urban: 3.9; 95% CI: 2.8–5.3; *p* < 0.001; rural: 4.1; 95% CI: 3.3–5.2; *p* < 0.001). Currently married individuals were approximately three and two times as likely to be overweight/obese compared to never married individuals, respectively, in the urban and rural areas (AOR: Urban: 3.1; 95% CI: 2.5–3.9; *p* < 0.001; rural: 2.2; 95% CI: 1.7–2.7; *p* < 0.001). Separated/Divorced/Widowed individuals were three and two times as likely to be overweight/obese compared to the never married individuals, respectively, in the urban and rural areas (AOR: Urban: 3.1; 95% CI: 2.5–3.9; *p* < 0.001; rural: 2.2; 95% CI: 1.7–2.7; *p* < 0.001). Residents of Mymensingh (AOR: Urban: 0.6; 95% CI: 0.4–0.8; *p* = 0.003; rural: 0.7; 95% CI: 0.5–0.8; *p* = 0.01) and Sylhet (AOR: Urban: 0.6; 95% CI: 0.4–0.9; *p* < 0.001; rural: 0.7; 95% CI: 0.5–0.9; *p* < 0.001) were less likely to be overweight/obese compared to the residents of Barisal division. Residents in the Rangpur division were less likely to be overweight/obese than those of the Barisal division in the rural area (AOR: 0.8; 95% CI: 0.6–1.0; *p* = 0.023). Thus, a significant association was observed in the urban area.

## 4. Discussion

This study aimed to investigate the differences in prevalence and associated factors of underweight and overweight/obesity among Bangladeshi adults stratified by place of residence using BDHS 2017–2018 data. A higher prevalence of overweight and obesity was found in the urban area in most categories, and an opposite phenomenon was observed in the rural area for underweight. In both regions, being 30–49 years of age, female sex, being educated up to college or higher level, living in the richest household, being currently married, or being separated/divorced/widowed increased the odds of overweight/obesity. Residence in Mymensingh and Sylhet region was associated with decreased odds of being overweight/obesity in both urban and rural areas. On the other hand, being educated up to college or higher level, living in the richest household, and being currently married were associated with reduced odds of being underweight in both areas.

The prevalence of overweight/obesity was higher than underweight (Overweight/obesity vs. underweight: urban: 50.23% vs. 12.24%; rural: 35.96% vs. 19.34%). This validates the prediction that, by 2015, the prevalence of overweight/obesity will exceed underweight [20,21]. The prevalence of overweight/obesity was higher in the urban area and the prevalence of underweight was higher in the rural area. This could be due to the urban area’s obesogenic environment (i.e., greater access to junk foods, inadequate space for physical activity, increased screen time) [22]. Osmani et al. found that food insecurity was higher in rural Bangladesh than in urban areas [23]. This could be one possible explanation behind the higher burden of being underweight in rural areas.

The highest prevalence of underweight was observed among the 30–49-year-old individuals. In the urban and rural areas, the highest odds of overweight/obesity were observed among this age group compared to 18–29-year-old participants. The classical ‘inverted U-shaped pattern’ of high BMI was observed in the overall adult population of Bangladesh [13] and in India, Nepal, and China [24,25,26]. This age group is at higher risk of developing non-communicable diseases due to the higher burden of high BMI and should be brought under public health intervention programs. Addressing non-communicable disease risk factors among the younger age group helps to prevent premature mortality and morbidity.

Although no significant association was observed between sex and underweight, a significant association was observed between sex and overweight/obesity in urban and rural areas. Females had higher odds of being overweight/obese than their male counterparts. This is a consistent finding regarding sex-wise distribution of overweight/obesity in developing countries [27,28]. This finding reiterates the need for targeted intervention focusing on females [13].

Similarly, like previous studies in both regions, the odds of overweight/obesity increased with increasing educational level and household wealth index [13,29,30,31]. In the case of underweight, the opposite trend was seen. In low- and middle-income countries like Bangladesh, many people with higher education and from rich backgrounds tend to live sedentary lifestyles. Also, they consume more energy-dense, nutrient-poor food. As a result, they gain more weight compared to their counterparts with an active lifestyle. On the other hand, perhaps due to food insecurity, the prevalence of underweight was higher among participants from the poorest households and those who received no formal education [13,29,30,31].

In the urban area, current working status was associated with decreased odds of underweight. No such association was observed in the rural area. This might be due to higher income in the urban area for the working class, and the fact that they are more food secure [22]. On the other hand, current working status was significantly associated with overweight/obesity in rural areas. Further exploration is needed to identify the reason behind this regional difference [32].

In rural Sylhet, Mymensingh, and Rangpur, the odds of overweight/obesity were also lower than in rural Barisal. Rangpur region has the highest prevalence of poverty in Bangladesh (47.23%), followed by Mymensingh division (32.8%) [33,34]. These regional differences in the odds of overweight and obesity must be explored. Nevertheless, it should be noted that in almost all the divisions of Bangladesh, the prevalence of overweight and obesity was more than 30% (except rural Mymensingh and rural Sylhet), indicating an ongoing nutritional transition.

In rural and urban areas, currently, married individuals had significantly lower odds of underweight and higher odds of overweight/obesity than the never married individuals. In the case of separated/divorced/widowed, higher odds were observed for overweight/obesity only. This was previously reported in Bangladesh [13,35]. It is hypothesized that in the presence of a spouse, eating habits are influenced, which leads to an increase in weight [36]. Public health promotion programs in Bangladesh should focus on ever married individuals.

In the last decade, Bangladesh has shown impressive achievement in reducing undernutrition among children and women [37]. On the other hand, the achievement in addressing the burden of overweight/obesity is limited [20,21]. Recently, a study showed that in Bangladesh, the urban–rural BMI gap among children and adolescents, notable in 1990, has significantly narrowed from 1990 to 2020 due to a more pronounced increase in BMI in rural areas. This trend has shifted the BMI of rural children from underweight towards normal or overweight thresholds [38]. The high burden of overweight/obesity is a barrier to the country’s ongoing effort to reduce the burden of non-communicable diseases. The nutrition interventions are entirely focused on underweight rather than overweight/obesity [20]. A multisectoral approach and translating the lessons learnt from other successful public health programs including the undernutrition prevention program are required to address the rising burden of overweight/obesity.

The strengths and weaknesses of the study warrant discussion. The study utilized a nationally representative sample. As a result, the findings of this study can be generalized in the context of Bangladesh. The study also utilized standard questionnaires that were validated in the context of Bangladesh. Calibrated instruments were also used. However, the study has notable limitations. This is a cross-sectional study and the temporal relationship between the exposure and the outcomes could not be established. Several covariates, including diet and physical activity, could not be adjusted because of the unavailability of data. Finally, BDHS 2014 did not have information on the participants’ medical histories, including any chronic or acute illnesses they may have had. Consequently, the findings of this study have not been adjusted for the potential influence of pre-existing health conditions or disease history.

## 5. Conclusions

This study found a high prevalence of overweight/obesity in Bangladesh’s urban and rural areas. Approximately one in two individuals in urban areas and one in three in rural areas were either overweight or obese. The prevalence of underweight was 12.24% and 19.34% in urban and rural areas, respectively. In both urban and rural areas, individuals aged 30–49 years, female sex, being educated up to college or higher level, living in the richest household, and being currently married, or being separated/divorced/widowed were at risk of developing overweight/obesity. These groups should be brought under health-promotion programs to curb the rising burden of overweight/obesity. On the other hand, underweight prevention programs should focus on individuals with no formal education, living in the poorest households, and residents of rural Mymensingh division.

## Figures and Tables

**Table 1:** Sample characteristics according to prevalence of different categories body mass index in urban and rural regions, BDHS 2017–2018.

Variables	Urban (n = 3412)						Rural (n = 9047)					
	Underweight		Normal Weight		Overweight/Obesity		Underweight		Normal Weight		Overweight/Obesity	
	n	%	n	%	n	%	n	%	n	%	n	%
**Age (in Years)**												
18–29	66	18.7	153	43.3	134	38.0	181	21.7	420	50.2	235	28.1
30–49	168	9.9	614	36.3	910	53.8	695	17.3	1820	45.3	1506	37.5
50–69	155	13.6	420	36.7	569	49.7	677	20.7	1431	43.8	1160	35.5
70+	28	12.5	93	42.1	100	45.4	197	21.4	373	40.5	351	38.1
**Sex**												
Male	222	14.2	682	43.6	659	42.2	871	22.0	1947	49.1	1147	28.9
Female	195	10.6	599	32.4	1054	57.1	879	17.3	2097	41.3	2106	41.4
**Education**												
No Formal Schooling	110	16.2	294	43.3	275	40.5	690	27.1	1212	47.6	645	25.3
Primary	142	15.4	393	42.8	385	41.8	548	19.5	1290	45.8	975	34.7
Secondary	101	9.5	382	36.0	580	54.6	359	14.1	1069	41.9	1121	44.0
College and Higher	65	8.7	211	28.1	475	63.2	153	13.4	473	41.6	512	45.0
**Wealth Index**												
Poorest	51	24.2	108	51.2	52	24.6	616	28.5	1046	48.5	497	23.0
Poorer	46	20.0	111	47.9	74	32.1	498	22.7	1098	49.9	603	27.4
Middle	75	16.6	200	44.4	176	39.0	355	16.9	965	45.9	781	37.2
Richer	120	13.1	402	43.6	399	43.3	198	12.9	627	40.8	711	46.3
Richest	125	7.8	460	28.8	1013	63.4	82	7.8	307	29.2	661	62.9
**Current Working Status**												
No	164	11.5	440	30.9	819	57.6	614	18.2	1436	42.6	1321	39.2
Yes	253	12.7	841	42.3	894	45.0	1136	20.0	2608	46.0	1932	34.0
**Division of Residence**												
Barisal	14	11.2	44	35.2	67	53.6	102	18.0	258	45.6	206	36.4
Chattogram	84	13.6	226	36.6	308	49.8	212	13.9	634	41.6	680	44.6
Dhaka	153	10.7	522	36.5	754	52.8	322	20.8	635	41.0	593	38.3
Khulna	39	11.3	122	35.0	187	53.7	192	16.2	500	42.3	491	41.5
Mymensingh	25	14.9	74	43.8	70	41.4	232	27.4	394	46.4	223	26.3
Rajshahi	45	12.8	146	41.2	163	46.1	276	19.5	671	47.3	472	33.3
Rangpur	28	12.6	85	38.0	110	49.5	260	20.2	636	49.5	390	30.3
Sylhet	28	19.8	62	42.8	54	37.5	154	23.1	315	47.3	198	29.7
**Marital Status**												
Never Married	96	23.1	195	47.1	123	29.8	223	26.4	427	50.6	195	23.1
Currently Married	272	10.1	978	36.1	1459	53.8	1305	17.9	3191	43.7	2812	38.5
Separated/Divorced/Widowed	50	17.2	108	37.3	131	45.5	222	24.8	426	47.7	246	27.5

**BDHS:** Bangladesh Demographic and Health Survey.

**Table 2:** Factors associated with underweight in both urban and rural area, BDHS 2017–2018.

Variables	Urban		Rural	
	AOR (95% CI)	*p*-Value	AOR (95% CI)	*p*-Value
**Age (in Years)**				
18–29	Ref		Ref	
30–49	0.6 (0.4–0.8)	0.001	0.8 (0.7–1.0)	0.094
50–69	0.8 (0.6–1.1)	0.104	1.1 (0.9–1.3)	0.521
70+	0.7 (0.4–1.0)	0.08	1.2 (0.9–1.6)	0.208
**Education**				
No Formal Schooling	Ref		Ref	
Primary	1.0 (0.8–1.3)	0.887	0.8 (0.7–0.9)	0.001
Secondary	0.8 (0.6–1.0)	0.094	0.7 (0.5–0.8)	<0.001
College and Higher	0.6 (0.4–0.9)	0.019	0.6 (0.5–0.8)	<0.001
**Wealth Index**				
Poorest	Ref		Ref	
Poorer	0.9 (0.6–1.4)	0.768	0.8 (0.7–0.9)	0.007
Middle	0.8 (0.6–1.2)	0.254	0.7 (0.6–0.8)	<0.001
Richer	0.6 (0.4–0.8)	0.001	0.6 (0.5–0.7)	<0.001
Richest	0.5 (0.4–0.8)	0.001	0.5 (0.3–0.6)	<0.001
**Current Working Status**				
No	Ref			
Yes	0.8 (0.7–1.0)	0.045	Not included in the final model	
**Division of Residence**				
Barisal	Ref		Ref	
Chattogram	1.3 (0.9–2.1)	0.178	0.9 (0.7–1.2)	0.449
Dhaka	1.2 (0.8–1.8)	0.457	1.3 (1.0–1.8)	0.084
Khulna	1.2 (0.8–1.8)	0.479	1.1 (0.8–1.4)	0.666
Mymensingh	1.2 (0.7–1.9)	0.482	1.4 (1.1–1.8)	0.018
Rajshahi	1.0 (0.7–1.6)	0.83	1.1 (0.8–1.5)	0.479
Rangpur	1.0 (0.6–1.6)	0.958	1.0 (0.7–1.3)	0.865
Sylhet	1.4 (0.9–2.2)	0.124	1.2 (0.9–1.6)	0.163
**Marital Status**				
Never Married	Ref		Ref	
Currently Married	0.6 (0.4–0.7)	<0.001	0.7 (0.5–0.8)	<0.001
Separated/Divorced/Widowed	0.8 (0.5–1.2)	0.218	0.8 (0.6–1.1)	0.153

AOR: Adjusted Odds Ratio; BDHS: Bangladesh Demographic and Health Survey; CI: Confidence Interval.

**Table 3:** Factors associated with overweight and obesity in both urban and rural areas, BDHS 2017–2018.

Variables	Urban		Rural	
	AOR (95% CI)	*p*-Value	AOR (95% CI)	*p*-Value
**Age (in Years)**				
18–29	**Ref**		**Ref**	
30–49	1.4 (1.1–1.9)	0.004	1.5 (1.2–1.8)	<0.001
50–69	1.3 (1.0–1.7)	0.025	1.4 (1.2–1.8)	0.001
70+	1.2 (0.8–1.7)	0.344	1.4 (1.1–1.8)	0.01
**Sex**				
Male	**Ref**		**Ref**	
Female	1.7 (1.4–2.0)	<0.001	2.0 (1.8–2.3)	<0.001
**Education**				
No Formal Schooling	**Ref**		**Ref**	
Primary	1.2 (1.0–1.5)	0.071	1.3 (1.1–1.5)	<0.001
Secondary	1.5 (1.2–1.9)	<0.001	1.6 (1.4–1.9)	<0.001
College and Higher	2.1 (1.7–2.7)	<0.001	1.7 (1.4–2.1)	<0.001
**Wealth Index**				
Poorest	**Ref**		**Ref**	
Poorer	1.3 (0.9–1.8)	0.22	1.1 (1.0–1.3)	0.176
Middle	1.7 (1.2–2.3)	0.002	1.6 (1.4–1.9)	<0.001
Richer	1.9 (1.4–2.5)	<0.001	2.1 (1.8–2.6)	<0.001
Richest	3.9 (2.8–5.3)	<0.001	4.1 (3.3–5.2)	<0.001
**Current Working Status**				
No	**Ref**		**Ref**	
Yes	0.9 (0.8–1.1)	0.183	1.2 (1.1–1.4)	0.002
**Division of Residence**				
Barisal	**Ref**		**Ref**	
Chattogram	0.8 (0.6–1.1)	0.212	1.0 (0.8–1.3)	0.954
Dhaka	0.8 (0.6–1.0)	0.081	0.9 (0.7–1.2)	0.591
Khulna	0.9 (0.7–1.2)	0.555	1.0 (0.8–1.3)	0.904
Mymensingh	0.6 (0.4–0.8)	0.003	0.7 (0.5–0.8)	0.001
Rajshahi	0.7 (0.5–1.0)	0.073	0.8 (0.6–1.0)	0.068
Rangpur	0.9 (0.7–1.3)	0.676	0.8 (0.6–1.0)	0.023
Sylhet	0.6 (0.4–0.9)	0.006	0.7 (0.5–0.9)	0.002
**Marital Status**				
Never Married	**Ref**		**Ref**	
Currently Married	3.1 (2.5–3.9)	<0.001	2.2 (1.7–2.7)	<0.001
Separated/Divorced/Widowed	2.4 (1.7–3.4)	<0.001	1.4 (1.1–1.9)	0.013

AOR: Adjusted Odds Ratio; BDHS: Bangladesh Demographic and Health Survey; CI: Confidence Interval.

## Data Availability

The de-identified data of BDHS 2017–2018 are available online: https://dhsprogram.com/data/dataset/Bangladesh_Standard-DHS_2017.cfm?flag=0 (accessed on 10 October 2021). following proper procedure.

## Appendix A

**Table A1:** Comparison of WHO-recommended BMI cut-off values with Asia Specific BMI cut-off values.

BMI Categories	WHO-Cut Off	Asia-Specific Cut Off
Underweight	<18.5	<18.5
Normal BMI	18.5 to <25.0	18.5 to <23.0
Overweight	25.0 to 30.0	23.0 to <27.5
Obesity	≥30.0	≥27.5

BMI: Body Mass Index; WHO: World Health Organization.

**Table A2:** Differences in the prevalence of underweight between urban and rural areas according to categories, BDHS 2017–2018.

Variables	Prevalence (%)		Differences (%)	95% CI, LL	95% CI, UL	*p* Value
	Urban	Rural				
**Age (in Years)**						
18–29	18.7	21.7	−2.9	−7.7	1.8	0.228
30–49	9.9	17.3	−7.3	−9.4	−5.3	<0.001
50–69	13.6	20.7	−7.1	−10.1	−4.2	<0.001
70+	12.5	21.4	−8.9	−15.0	−2.8	0.004
**Sex**						
Male	14.2	22	−7.7	−10.3	−5.2	<0.001
Female	10.6	17.3	−6.7	−8.7	−4.8	<0.001
**Education**						
No Formal Schooling	16.2	27.1	−10.9	−14.5	−7.2	<0.001
Primary	15.4	19.5	−4.1	−7.2	−1.0	0.009
Secondary	9.5	14.1	−4.6	−7.1	−2.2	<0.001
College and Higher	8.7	13.4	−4.7	−7.9	−1.5	0.004
**Wealth Index**						
Poorest	24.2	28.5	−4.3	−9.5	0.9	0.104
Poorer	20	22.7	−2.6	−7.6	2.3	0.294
Middle	16.6	16.9	−0.3	−4.3	3.7	0.884
Richer	13.1	12.9	0.2	−3.2	3.5	0.917
Richest	7.8	7.8	0.0	−2.7	2.7	0.996
**Current Working Status**						
No	11.5	18.2	−6.7	−9.1	−4.3	<0.001
Yes	12.7	20	−7.3	−9.4	−5.1	<0.001
**Division of Residence**						
Barisal	11.2	18	−6.8	−11.7	−1.9	0.007
Chittagong	13.6	13.9	−0.3	−3.9	3.4	0.89
Dhaka	10.7	20.8	−10.1	−15.1	−5.0	<0.001
Khulna	11.3	16.2	−4.9	−8.5	−1.3	0.008
Mymensingh	14.9	27.4	−12.5	−17.9	−7.1	<0.001
Rajshahi	12.8	19.5	−6.7	−11.8	−1.6	0.01
Rangpur	12.6	20.2	−7.7	−12.4	−2.9	0.002
Sylhet	19.8	23.1	−3.3	−9.2	2.6	0.268
**Marital Status**						
Never Married	23.1	26.4	−3.2	−8.9	2.4	0.256
Currently Married	10.1	17.9	−7.8	−9.5	−6.1	<0.001
Separated/Divorced/Widowed	17.2	24.8	−7.7	−12.8	−2.5	0.004

BDHS: Bangladesh Demographic and Health Survey; CI: Confidence Interval; LL: Lower Limit; UL: Upper Limit.

**Table A3:** Differences in the prevalence of overweight/obesity between urban and rural areas according to categories, BDHS 2017–2018.

Variables	Prevalence (%)		Differences (%)	95% CI, LL	95% CI, UL	*p* Value
	Urban	Rural				
**Age (in Years)**						
18–29	38	28.1	9.9	2.5	17.2	0.009
30–49	53.8	37.5	16.3	12.8	19.8	<0.001
50–69	49.7	35.5	14.2	10.1	18.2	<0.001
70+	45.4	38.1	7.3	−1.6	16.3	0.108
**Sex**						
Male	42.2	28.9	13.2	9.5	17.0	<0.001
Female	57.1	41.4	15.6	12.7	18.5	<0.001
**Education**			0.0	0.0	0.0	<0.001
No Formal Schooling	40.5	25.3	15.2	10.8	19.6	<0.001
Primary	41.8	34.7	7.2	3.0	11.3	0.001
Secondary	54.6	44	10.6	6.2	15.0	<0.001
College and Higher	63.2	45	18.2	12.9	23.5	<0.001
**Wealth Index**						
Poorest	24.6	23	1.6	−4.6	7.7	0.618
Poorer	32.1	27.4	4.7	−2.0	11.4	0.169
Middle	39	37.2	1.8	−2.9	6.5	0.445
Richer	43.3	46.3	−3.0	−8.7	2.8	0.31
Richest	63.4	62.9	0.5	−4.5	5.4	0.858
**Current Working Status**						
No	57.6	39.2	18.4	14.7	22.0	<0.001
Yes	45	34	10.9	7.8	14.1	<0.001
**Division of Residence**						
Barisal	53.6	36.4	17.2	8.1	26.4	<0.001
Chittagong	49.8	44.6	5.3	−1.5	12.0	0.127
Dhaka	52.8	38.3	14.5	7.8	21.2	<0.001
Khulna	53.7	41.5	12.1	6.3	18.0	<0.001
Mymensingh	41.4	26.3	15.1	5.8	24.5	0.002
Rajshahi	46.1	33.3	12.9	5.3	20.4	0.001
Rangpur	49.5	30.3	19.2	10.3	28.0	<0.001
Sylhet	37.5	29.7	7.8	1.1	14.5	0.024
**Marital Status**						
Never Married	29.8	23.1	6.8	0.7	12.8	0.028
Currently Married	53.8	38.5	15.4	12.5	18.3	<0.001
Separated/Divorced/Widowed	45.5	27.5	18.0	11.8	24.2	<0.001

BDHS: Bangladesh Demographic and Health Survey; CI: Confidence Interval; LL: Lower Limit; UL: Upper Limit.
